# Is the Six-Minute Walk Test the Key to Boost Postoperative Clinical Outcomes in Cardiac Surgery?

**DOI:** 10.21470/1678-9741-2024-0241

**Published:** 2025-11-17

**Authors:** Isadora Salvador Rocco, Walter José Gomes, Caroline Bublitz, Alexandra Ribeiro Monte Sião, Nelson A. Hossne Junior, Solange Guizilini

**Affiliations:** 1 Cardiovascular Surgery Discipline, Escola Paulista de Medicina, Universidade Federal de São Paulo, São Paulo, São Paulo, Brazil; 2 Cardiology Postgraduation Program, Escola Paulista de Medicina, Universidade Federal de São Paulo, São Paulo, São Paulo, Brazil

**Figure:**
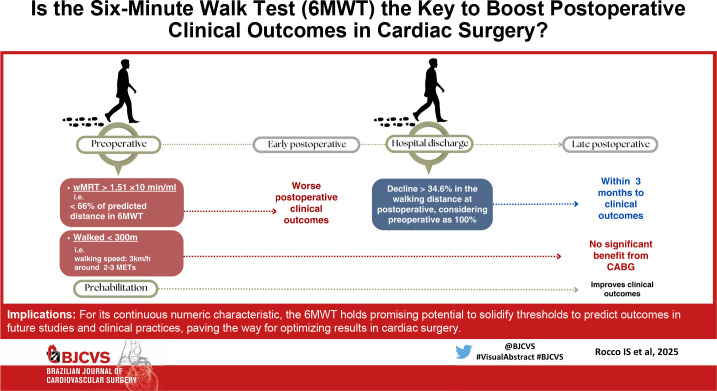
CABG=coronary artery bypass grafting; METs=metabolic costs; wMRT=work corrected mean response time.

**Table:** 

Abbreviations, Acronyms & Symbols				
6MWT	= Six-minute walk test		METs	= Metabolic costs
CABG	= Coronary artery bypass grafting		OMT	= Optimal medical therapy
CPR	= Cardiopulmonary resuscitation		STICH	= Surgical Treatment for Ischemic Heart Failure
ECC	= External chest compression		wMRT	= Work corrected mean response time
LV	= Left ventricular			

The role of the six-minute walk test (6MWT) has expanded to become a valuable tool for evaluating submaximal exercise capacity in patients with cardiovascular diseases, serving as an independent predictor for adverse events and mortality^[1,2]^. The 6MWT unveils the threshold at which symptoms may manifest during activities, thereby delineating functional limitations. Such information is a cornerstone for the understanding of a disease's impact and, therefore, to predict clinical courses. Greater performance during the 6MWT suggests better preservation of peripheral musculature and reveals the presence of adaptive mechanisms that overcome underlying oxygen delivery challenges of cardiovascular diseases^[3,4]^. On the other hand, a poor performance during the test unveils circulatory deficits followed by consequences in other systems, leading to a worse prognosis^[4,5]^.

Findings stemming from the seminal Surgical Treatment for Ischemic Heart Failure (STICH) trial revealed a pivotal insight into the risk stratification for coronary artery bypass grafting (CABG)^[6]^. The STICH trial compared CABG with optimal medical therapy (OMT) in patients with advanced coronary artery disease and severe left ventricular (LV) dysfunction. After a median of approximately 10 years of follow-up, patients randomized to CABG had lower all-cause and cardiovascular mortality compared with those with OMT. A further substudy of the STICH trial reported that baseline 6MWT distance predicted mortality during late follow-up in the STICH trial. Patients unable to walk 300 meters had higher mortality during the first 60 days with CABG and no significant benefit from CABG during total follow-up^[7]^. These observations suggest that patients with ischemic LV dysfunction and poor exercise capacity have increased early risk, while those with better exercise capacity have improved survival with CABG.

This brought to debate that patients' clinical condition and fitness matter for the results of surgery, emphasizing the important role of the 6MWT in determining potential surgical outcomes. Likewise, our research group made a significant advance to this field through an in-depth investigation into the physiological response during the transition from rest to effort in the 6MWT, thereby establishing its predictive ability for early outcomes following CABG^[8]^. The same findings were observed for patients in the preoperative period of valve surgery, where poor performance of 6MWT was associated with worse results following surgery^[9]^.

Despite these findings around predictive abilities of the 6MWT, it remains conspicuously absent from the standard preoperative assessment protocols for perioperative management. Its integration into routine evaluation practices and well-established risk stratification scores within the cardiac surgery domain has been notably lacking. Recent updates in risk models, such as the European System for Cardiac Operative Risk Evaluation 3, emphasizes a proactive approach to enhance predictive ability following cardiac surgery^[10]^. Integrating responses to the 6MWT into these models holds promise for refining risk stratification. By capturing a patient's physiological response to submaximal effort, this dynamic evaluation could provide valuable insights and identify patients in need for prehabilitation interventions, potentially improving the overall success and safety of cardiac surgeries. Nevertheless, studies in this field are necessary to comprehensively assess the safety of conducting the 6MWT preoperatively.

Beyond its predictive role, the 6MWT can quantify the acute impact of cardiac procedure in functional capacity during the postoperative period. Studies have shown an inherent drop on the distance walked at hospital discharge compared to the preoperative period that varies around 12 to 17%^[11,12]^. When the 6MWT is applied earlier, right after intensive care unit discharge around the fifth postoperative day, this fall can reach over 30%^[13]^. Recent findings of our group have uncovered that a decline exceeding 34.6% is associated with unfavorable midterm outcomes following CABG. Although these assumptions lack strong evidence, it brings to light the necessity to investigate the potential role of systematically evaluating 6MWT performance during both pre and postoperative periods.

Postoperative assessment using the 6MWT at hospital discharge provides valuable prognostic information, facilitating effective screening for outpatient care. Beyond assessing mere walking distance, it serves as a measure of speed, which holds significance in tailoring postoperative exercise prescription. Additionally, it unveils the level of metabolic costs (METs) at which patients experience a comfortable walking speed. For instance, performing 300 meters in six minutes is equivalent to a walking speed of 3 km/hour, which corresponds to a metabolic expenditure of two to three METs. This information is crucial for gauging functional limitations and monitoring efficacy of therapeutics, given that an increase in walking speed, such as reaching 4 - 5 km/hour, may signify a gain of one MET, indicative of 12% improvement in life expectancy^[14]^.

Literature in this field has established a reasonable causal relationship between impaired functional capacity and mortality following cardiac surgery^[15]^. Preoperative 6MWT serves as a reliable indicator of the patient's physical reserve and, therefore, predicts the ability to withstand the physiological demands of surgery and subsequent recovery. Moreover, exercise intolerance impacts postoperative strategies of enhanced recovery, such as early walking, exposing patients to a higher risk of complications and poor outcomes. Arthur et al.^[16]^ demonstrated that a multidimensional preoperative intervention significantly reduced hospital stay and improved quality of life in low-risk patients undergoing elective CABG. These assumptions support the importance of integrating exercise training into the preoperative care to optimize functional status and improve overall prognosis and surgical outcomes^[17,18]^. A comprehensive approach of multimodal exercise modalities including aerobic and resistance exercises, especially inspiratory muscle training^[18]^, has been recommended to enhance readiness and improve surgical outcomes.

Finally, incorporating the 6MWT during the perioperative period of cardiac surgery not only enhances risk stratification, but also contributes significantly to the decision-making process, ultimately aggregating results to the surgical procedure. Therefore, the 6MWT should be applied in both pre and postoperative clinical contexts, during the decision-making process that defines patients eligible for cardiac surgery and early after surgery at hospital discharge, followed by serial assessments in the outpatient postoperative setting. For its continuous numeric characteristic, the 6MWT holds promising potential to solidify thresholds to predict outcomes in future studies and clinical practices, paving the way for optimizing results in cardiac surgery.

## Data Availability

The authors declare that data sharing is not applicable to this article as no new data were created or analyzed

## Artificial Intelligence Usage

The authors declare the use of ChatGPT and DALL-E exclusively for the development of the thorax, which is one of the components of the central figure. The content produced by the artificial intelligence tool was revised and edited by the authors as necessary, and they take full responsibility for the content to be published.
